# Brain Responses to Food Odors Associated With BMI Change at 2-Year Follow-Up

**DOI:** 10.3389/fnhum.2020.574148

**Published:** 2020-10-08

**Authors:** Pengfei Han, Hong Chen, Thomas Hummel

**Affiliations:** ^1^The Key Laboratory of Cognition and Personality, Ministry of Education, Chongqing, China; ^2^Faculty of Psychology, Southwest University, Chongqing, China; ^3^Interdisciplinary Center Smell and Taste, Department of Otorhinolaryngology, TU Dresden, Dresden, Germany

**Keywords:** brain activation, food odor, fMRI, BMI change, 2-year follow-up

## Abstract

The understanding of food cue associated neural activations that predict future weight variability may guide the design of effective prevention programs and treatments for overeating and obesity. The current study investigated the association between brain response to different food odors with varied energy density and individual changes of body mass index (BMI) over 2 years. Twenty-five participants received high-fat (chocolate and peanut), low-fat (bread and peach) food odors, and a nonfood odor (rose) while the brain activation was measured using functional magnetic resonance imaging (fMRI). BMIs were calculated with participant’s self-reported body weight and height collected at the time of the fMRI scan and again at 2 years later. Regression analyses revealed significant negative correlations between BMI increase over 2 years and brain activation of the bilateral precuneus and the right posterior cingulate cortex (PCC) in response to high-fat vs. low-fat food odors. Also, brain activation of the right supplementary motor area (SMA) in response to food vs. non-food odor was negatively correlated to subsequent BMI increase over 2 years. Taken together, the current findings suggest that individual differences in neural responsivity to (high calorie) food odors in brain regions of the default mode and motor control network serve as a neural marker for future BMI change.

## Introduction

Bodyweight variability in humans is usually regulated by many factors including diet and lifestyle (Mozaffarian et al., 2011). Bodyweight gain is the result of a long-term imbalance between energy consumption and energy expenditure, which is mainly triggered by excessive intake of high-calorie food and the dysfunction of the biological system for body weight regulation. External appetitive food cues are important drivers for eating behavior, with higher food cue reactivity and food craving predict weight gain (Boswell and Kober, 2016). Another key risk factor for weight gain is the motivation to eat, or food reinforcement (Epstein et al., 2014).

In recent years, brain activity as biological predictors for behavioral changes, especially in the area of food consumption and weight change, has been increasingly noted (Giuliani et al., 2018). Using functional magnetic resonance imaging (fMRI) and food cue-based stimulation paradigms, previous studies have found correlations between brain reactivity to food cues and long-term body weight change. For example, elevated brain activation of the reward-related regions (e.g., striatum, orbitofrontal cortex, anterior cingulate cortex, insula) in responses to food pictures (Yokum et al., 2011; Demos et al., 2012; Murdaugh et al., 2012; Hermann et al., 2019), food advertisements (Yokum et al., 2014), abstract cues that relate to palatable food (Stice et al., 2015) predicted later weight gain (Yokum et al., 2011, 2014; Demos et al., 2012; Stice et al., 2015) or less success in a weight-loss process (Murdaugh et al., 2012; Hermann et al., 2019). Apart from visual food cue, other studies have shown that an elevated reward brain activation in response to tastes of palatable food predicted future weight gain (Geha et al., 2013; Winter et al., 2017). Thus, the hyper brain responsivity involved in the incentive valuation of external food cues may act as a vulnerability factor that promotes food overconsumption and lead to long-term weight gain (Stice and Yokum, 2016). However, one recent research applying a bootstrapping sampling approach had suggested that only little reliable evidence regarding stronger reward brain responses to food images or food tastes predicted future weight gain (Stice and Yokum, 2018). Rather, the lower brain activation of the pre-supplementary motor area (SMA) in response to an oral tasting of high-fat/low-sugar milkshake and the increased activation of the precentral gyrus/Rolandic operculum in responses to palatable food images were moderately reliable predictors for future weight gain (Stice and Yokum, 2018). Noted that dietary macronutrients have different contributions to food intake and body weight, with dietary fat as a more potent factor related to excessive energy consumption and weight gain (Hu et al., 2018). A recent study investigated the neural response to a milkshake with varying levels of sugar or fat had found that individuals with greater activation in the postcentral gyrus, insula, and medial prefrontal cortex in response to high-fat vs. low-fat milkshake gained more weight in the later period (Yokum and Stice, 2019).

Appetitive food odors are powerful food stimuli that can trigger dopaminergic brain responses, and increase the motivation to eat (Boesveldt and de Graaf, 2017). Specifically, food odors steer appetite (Zoon et al., 2016; Proserpio et al., 2019) and craving for specific foods (Larsen et al., 2012), which largely determine food preference, selection, and consumption. Moreover, food odors can signal information including the calorie density, taste quality, and specific macronutrient content of the odor related food. Those odor-taste or odor-nutrient associations are developed through the learning process during repeated food consumption (Stevenson and Boakes, 2004). Therefore, food odors can be distinguished according to the taste quality or nutrient content (e.g., high or low fat; Boesveldt and de Graaf, 2017; Morquecho-Campos et al., 2019). Brain imaging studies showed that food odors compared to non-food odors mainly activate the reward-related brain regions (Bragulat et al., 2010; Eiler et al., 2012; Sorokowska et al., 2017). However, little research had been done on exploring the food odor elicited brain responsivity and future body weight changes.

The objective of the current study was to investigate the correlation between individual brain responses to food odors and future weight change. Using a sub-sample from a previous study (Han et al., 2020), brain activation in response to food (varied in fat content and sweetness) and nonfood odors were assessed among healthy adult participants. Body mass index (BMI) at the time of the fMRI test and again at 2-year later were calculated using self-reported height and body weight. Based on recent studies addressing similar research questions (Yokum and Stice, 2019), we tentative hypothesized that individual brain response to food odors correlate to subsequent BMI changes. Specifically, we hypothesized that greater activation of the taste and motor-related brain regions in response to high-fat vs. low-fat food odors would be associated with a larger BMI increase over 2 years.

## Materials and Methods

### Participants

Of the 38 right-handed participants included in the initial study (Han et al., 2020), 25 of them were contacted *via* email or social networking tools (WeChat or Facebook) 2-years later and were included in the current analyses. Normal olfactory functions of them were ascertained using the threshold and identification test of the “Sniffin’ Sticks” battery (Hummel et al., 1997). Also, all participants’ behavior characteristics were measured using the Three-Factor Eating Questionnaire (Stunkard and Messick, 1985). Female participants were not at any stage of pregnancy at the time of the MRI scan and 2-years later according to their self-report. However, one female participant gave birth in May 2019. Self-reported height and body weight were recorded at the baseline session and 2 years later. BMI was calculated for each participant. BMI is a sensitive measure of adiposity that is adjusted for variation in height. The characteristics of included participants were shown in Table 1. The study was conducted according to the Declaration of Helsinki and was approved by the Ethics Board of the University of Dresden Medical School (#EK22012018). All participants signed an informed consent form prior to the tests.

**Table 1:** Study participant characteristics (*N* = 25).

	M or %	SD	Range
Age	25.2	2.9	21–32
Sex			
Female	60%
Male	40%
BMI	
Baseline	21.5	1.7	18.5–26.7
At the 2-year follow-up	21.6	2.1	18.4–26.7
Sniffin’ Sticks test			
Threshold (phenyl ethanol)	9.7	2.9	4–16
Identification	13.3	1.2	11–16
TFEQ
Restraint	7.8	4	0–15
Disinhibition	6.2	3.2	0–11
Hunger level during fMRI	4.2	1.1	2–6

*Abbreviations: TFEQ, three-factor eating questionnaire. Hunger level was assessed using 7-point scales*.

### Odor Stimuli

Odors that signaling food items with varied fat content and taste qualities were chosen as food-related odor stimuli, including a high-fat sweet chocolate odor (Fragrance Resources, Hamburg, Germany; Product code 51615/3), a high-fat non-sweet peanut odor (Symrise, GmbH, KG; Product code 10464774/3), a low-fat sweet peach odor (Frey und Lau, Henstedt-Ulzburg, Germany; Product code P0606040), and a low-fat non-sweet bread odor (Fragrance Resources, Hamburg, Germany; Product code PG93193). Also, a rose-like odor (Takasago, Paris, France; Product code DG FLO 792A) was chosen as non-food olfactory stimuli. The odorants were diluted in propylene glycol to the following finalized concentrations to reach the proper and equal intensity according to daily life experiences: chocolate 1% (v/v) for chocolate odor; 20% (v/v) for peanut odor; 1% (v/v) for peach odor, 5% (v/v) for bread odor, and 0.5% (v/v) for rose odor.

### fMRI Paradigm

All participants were refrained from consuming food or drinks (except water) for 2 h before taking part in the experiment. On arrival and before the fMRI test, participants rated their hunger/fullness levels on a 9-point scale (from 0 very hungry to 9 very full). The hunger ratings were used to check compliance with the fasting instructions and were used as a covariate during the correlation analysis between brain activation and future body weight changes, as suggested by a previous study (Winter et al., 2017). For the fMRI testing, food and non-food odors were embedded in a constant flow rate of clean air (2 liters/min) and were delivered to the bilateral nostrils of the participants using a portable olfactometer (Sommer et al., 2012). We used an “ON-OFF” block design paradigm for odor stimulation. Each block lasts 25 s, including a 15-s “odor ON” period followed by a 20-s “odor OFF” baseline period. To minimize odor habituation, participants received the odor stimuli intermittently during the “odor ON” period, with 1-s of odorized air (e.g., chocolate, peach, peanut, bread, or rose) and 2-s of odorless air. During the “odor OFF” baseline period, participants received only odorless air. This design permitted an assessment of brain activation in responses to each specific odor type with airflow being subtracted out (Small et al., 2005). There were 10 repeated blocks in each functional run (for a single odor) with the time duration 5 min and 50 s. The order of administration of the five odors (five runs) was randomized among participants. The total time for the fMRI test was about 35 min.

After each run, participants verbally rated the intensity (0–10; “not perceived” to “very strongly perceived”), pleasantness (0–10; “extremely unpleasant” to “extremely pleasant”), sweetness (0–10; “not sweet at all” to “very sweet”) of the odor stimuli, and their desirability to eat food with a similar odor (0–10; “Do not want to eat at all” to “Want to eat very much”) *via* the intercom. The participants were asked to keep their head still during the fMRI scan and also the verbal reporting. Before the fMRI test, participants took part in a training session where they practiced the velopharyngeal closure which enables breathing only through the mouth (Kobal, 1981), and were instructed to use this technique during the fMRI scanning. This technique can effectively eliminate the influences of the nasal breathing cycle (inhalation and exhalation) on brain activations, and had been used in multiple previous fMRI studies with a similar setup (Kareken et al., 2004; Croy et al., 2014; Wallrabenstein et al., 2015; Han et al., 2020).

### Odor Ratings

To verify the significant differences regarding the energy density, taste quality, and major macronutrient content between the selected odors, participants completed an estimation task for the fat content and calorie density for the odor related food items after the fMRI session. We asked participants to rate the fat content and calorie density of the odor-associated food with the question “*Please estimate the fat content of the odor-associated food*,” and the question “*Please estimate the calorie density of the odor-associated food*,” respectively. Ratings were performed on a 9-point scale for macronutrient content (range from 1 = very low to 9 = a large amount) and calorie density (1 = very low-calorie density, to 9 = very high-calorie density). Notably, participants were not aware of the aforementioned rating task before and during the fMRI scan. This was meant to avoid biased brain activation results caused by any selective attention to such food attributes during the fMRI test.

### Imaging Data Acquisition and Preprocessing

We collected the whole-brain functional and structural images with an 8-channel head coil on a 3 Tesla MRI scanner (Siemens Sonata, Erlangen, Germany). For each participant, functional images were collected using a T2-weighted echo-planar imaging (EPI) sequence (TR = 2,500 ms; TE = 40 ms; Flip Angle = 90°; voxel size = 3*3*3.75 mm; Field of View, FoV = 192 × 192 mm). A high-resolution T1-weighted anatomical image was acquired using a standard MPRAGE (magnetization prepared rapid acquisition gradient echo) sequence (TR = 2,530 ms; TE = 2.34 ms; Field of View, FoV = 256 × 256 mm; voxel size = 1*1*1 mm).

fMRI data were analyzed using SPM12 (Statistical Parametric Mapping; Welcome Department of Cognitive Neurology, London, UK) run with MATLAB (version 2014a, MathWorks, MA, USA). For image preprocessing, we used the standard pipeline batch in (\spm12\batches\preproc_fmri.m). In short, the preprocessing included realignment to the first image of the first run and unwrapped, co-registration with anatomical image, segmentation and spatial normalization of the standard EPI MNI template, and spatial smoothing with an 8-mm full width at half maximum (FWHM) isotropic Gaussian kernel. Finally, we used the ArtRepair tool (version 4, Stanford University) to examine the post-preprocessing brain volumes to detect and repair the artifact volumes due to excessive head motion. Brain volumes with image-to-image motion larger than 0.5 mm/TR and total volumes repaired exceed 20% of the total volumes were excluded from the analysis. Data from 17 participants were repaired with no participant was excluded at this stage.

### Imaging Data Analysis

For the baseline period, we discarded the first 5 s due to the potential carry-over effect from the odor period. We performed the analysis in a two-step manner. First, we modeled the following contrasts for each participant: food > nonfood odor; high-fat > nonfood odor; low-fat > nonfood odor; high-fat > low-fat odor; low-fat > high-fat odor, sweet > savory odor, savory > sweet odor. Then, on the group level, we entered the contrasts from all individual participants into a linear regression model to explore the association between brain responses and 2-year BMI changes. Subjective hunger ratings and baseline BMIs were entered as covariates as suggested in previous studies (Winter et al., 2017).

Statistical analyses were performed on a whole-brain level. To control for multiple statistical testing within the entire brain, we maintained a cluster-level false-positive detection rate at *p* < 0.05 using an initial voxel-level threshold of *p* < 0.001 in combination with a cluster extent (k) empirically determined by 1,000 Monte Carlo simulations, using AlphaSim as implemented in the REST toolbox^1^ (Song et al., 2011). Simulation results indicated that a minimum cluster size of 66 contiguous voxels under the threshold of *p* < 0.001 would provide sufficient cluster-level Family-Wise Error correction of *p* < 0.05 across the whole brain. Mean responses (BOLD signals) of the significant clusters were extracted using the Marsbar toolbox. Specific brain regions were identified using the Automated Anatomical Labeling (AAL) toolbox (Tzourio-Mazoyer et al., 2002).

## Results

### Psychophysical Odor Ratings

**Figure 1:**
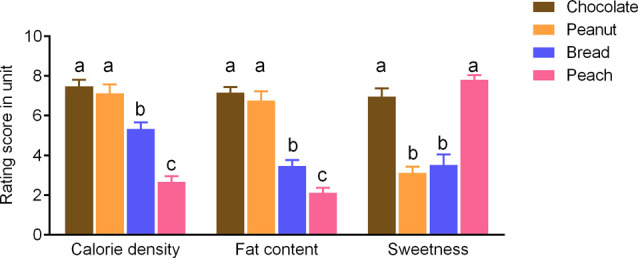
Rating scores of the sweetness levels and estimated fat content or calorie density for food odors). Values are means with error bars (SEM), with different lowercase letters indicate significant differences (*p* < 0.05).

Participants rated the chocolate and peanut odors as significantly higher in terms of the fat content and calorie density as compared to bread and peach odors (*p* < 0.05; Figure 1). On the other hand, the chocolate and peach odors were rated as sweeter compared to the peanut and bread odors (*p* < 0.05; Figure 1). There was no intensity rating difference between odors (*p* > 0.1).

### Brain Response to High-Fat vs. Low-Fat Food Odor Correlates to BMI Increase Over 2 Years

**Figure 2:**
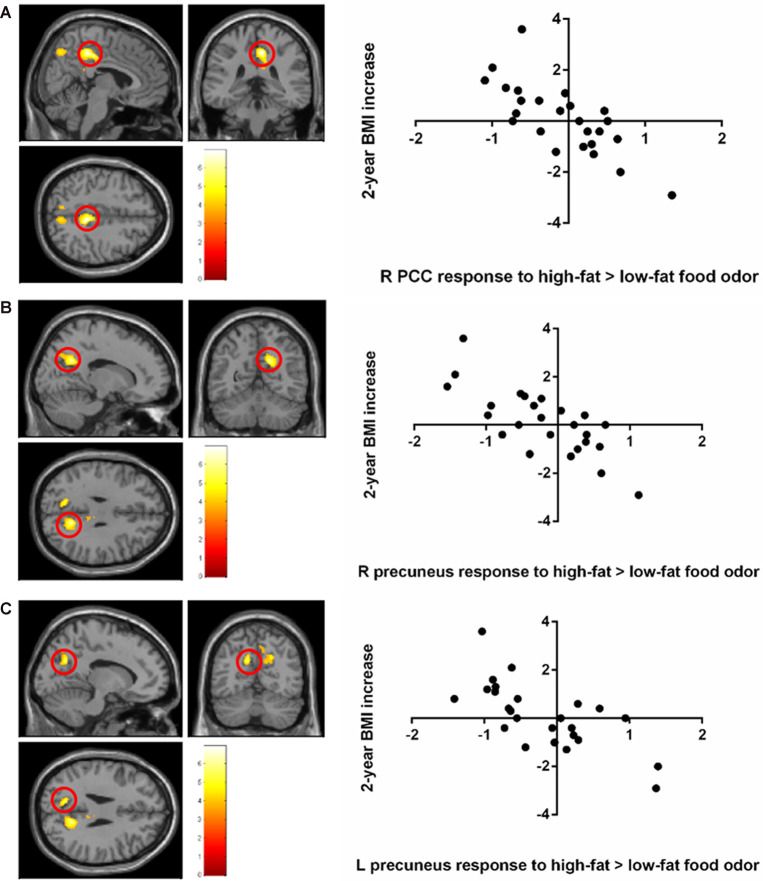
Negative correlation between the 2-year body mass index (BMI) increase and baseline brain responses to high-fat vs. low-fat food odors in **(A)** the posterior cingulate cortex (PCC), **(B)** the left precuneus, and **(C)** the right precuneus. Brain activation was significant with corrected *p* < 0.05 and was illustrated on a template provided in SPM12 (single_sub_T1.nni).

Stronger BOLD responses to the contrast of high-fat > low-fat food odors in the right posterior cingulate cortex (PCC; peak *T* = 6.92, *k* = 470, corrected *p* < 0.05; Figure 2A; Table 2) and the bilateral precuneus (right hemisphere peak *T* = 5.95, *k* = 462; left hemisphere peak *T* = 5.69, *k* = 145, corrected *p* < 0.05; Figures 2B,C; Table 2) predicted lower BMI increase over 2 years. There was no observed significant correlation between the brain response to low fat > high-fat food odors and future BMI increase.

**Table 2:** Negative correlations between baseline brain response to High-calorie > Low-calorie odors and food > nonfood odors and BMI change over 2 years.

	Cluster size	Peak T	pFWEcorr.	MNI coordinates (xyz)			Region (AAL)
High-fat > low-fat odor	470	6.92	<0.001	6	−36	44	Posterior cingulate cortex R
	462	5.95	<0.001	16	−56	30	Precuneus R
	145	5.69	0.046	−12	−64	28	Precuneus L
Food > non-food odor	121	6.13	0.069	14	15	54	Supplementary motor area R

*All reported results were significant at uncorrected *p* < 0.001 and cluster size of *k* > 66 contiguous voxels across the whole brain. Abbreviations: FWEcorr., Family-Wise Error corrected; AAL, Automated Anatomical Labeling; MNI, Montreal Neurological Institute*.

### Brain Response to Food vs. Nonfood Odor Correlates to BMI Increase Over 2 Years

**Figure 3:**
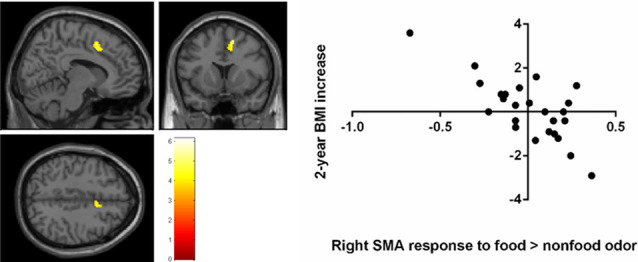
Negative correlation between the 2-year BMI increase and the baseline brain responses to food vs. nonfood odors in the right supplementary motor area (SMA) Brain activation were significant with corrected *p* < 0.05 and were illustrated on a template provided in SPM12 (single_sub_T1.nni).

A negative correlation was found between brain response to food > nonfood odors in the SMA and BMI increase over 2 years (cluster size = 121 voxels, peak *T* = 6.13, corrected *p* < 0.05; Table 2; Figure 3). There is no significant brain response to sweet vs. savory odors and BMI change.

### Correlation Between Psychometric Variables and BMI Increase Over 2 Years

No significant correlation was found between olfactory performance (odor threshold, odor identification scores), TFEQ scores, psychophysical odor ratings (pleasantness, intensity, sweetness, or desirability), and increases of BMI or body weight over the 2 years follow up (all *p* > 0.1).

## Discussion

In a group of healthy adult participants, individual variation of BMI changes over 2 years was related to the baseline brain responsivity to food odor cues. Specifically, a lower level of baseline brain activation in the bilateral precuneus and the right PCC in response to high-fat vs. low-fat food odors was correlated with BMI increase over 2 years. Both the precuneus and the PCC are key areas of the default mode network. The default mode network is active when participants are not focused on the external environment and attention is turned inward (Buckner et al., 2008). The precuneus was hypothesized to have a unifying function in self-mental representation and modulation of conscious processes (Cavanna and Trimble, 2006), and to play a role in olfactory related imagery or episodic memory retrieval (Plailly et al., 2012; Flohr et al., 2014). It had been argued that this region is related to self-referential processes, appetite control, or conscious suppression of food craving (Tuulari et al., 2015). For example, the precuneus have been implicated in evaluating the benefits of not eating compared with eating high-calorie palatable foods (Yokum and Stice, 2013), and assessing the healthiness of food items (Herwig et al., 2016). Stronger precuneus activation in response to palatable milkshake taste (Winter et al., 2017) or unhealthy food commercials (Gearhardt et al., 2020) was correlated to less weight gain over 3 years or less healthy food consumption, respectively. Oral taste of high vs. low-fat milkshake independent of sugar content involved in the PCC activation difference that predicts future weight loss (Yokum and Stice, 2019). In a recent study, Roux-en-Y gastric bypass surgery-induced decreased liking of high-fat/high-sugar food odors was accompanied by an increased precuneus activation (Zoon et al., 2018). Taken together, the activation of the precuneus in responses to high-fat food odors may be protective against future weight gain. However, other studies had shown the precuneus and PCC activation during imagination of eating (Yokum and Stice, 2013; Kiortsis et al., 2018) or reward/motivational processing of odors (Bragulat et al., 2008, 2010; Han et al., 2018b). Future work is necessary to elucidate the specific functioning mechanism of these regions behind the association between the activation during food cue processing and future weight changes.

Further, lower SMA activation in response to high-fat/low-sugar milkshake predicted later weight gain in adolescents (Stice and Yokum, 2018), which is in line with the current finding. The SMA had been frequently observed with food cue stimulation (Wagner et al., 2013), and was involved in cognitive reward control including food-related cues (Brandl et al., 2019). A recent meta-analysis of fMRI studies showed the recruitment of SMA activation when participants attempt to reduce their craving response to food cues (Han et al., 2018a). Besides, the SMA has been implicated in inhibitory processes (for meta-analyses, see Simmonds et al., 2008; Swick et al., 2011; Criaud and Boulinguez, 2013). To illustrate, Hollmann et al. (2012) found robust activation of the SMA during the downregulation of food desire using a cognitive reappraisal strategy compared to the “admit to food desire” condition. Stronger SMA activation was observed among successful vs. unsuccessful self-controllers during healthy food choices (van der Laan et al., 2014). An earlier study also showed increased SMA activation along with reduced pleasure during repeated food consumption (Small et al., 2001).

We did not observe significant activations of the reward-related brain regions (e.g., the striatum or insula) that predicted 2-year BMI or weight gain. A recent study using a bootstrap sampling approach had evaluated the reliability of the food-related neural responsivity as predictors for weight change among 10 bootstrap samples. An elevated response in the precentral gyrus/Rolandic operculum to images of appetizing foods turned out to be a moderately reliable predictor of future BMI gain (Stice and Yokum, 2018). However, the reward brain activation was less reliable (although one study found elevated responsivity of regions implicated in reward processing predicted future weight gain, see Geha et al., 2013). Our results further added to that by showing that brain activation of reward-related areas in response to appetitive food odors did not predict future BMI changes.

A major limitation of the current study was that important factors related to bodyweight alterations (e.g., regular physical activities, diet, stressors, metabolic alterations) were not followed during the 2-year follow-up, which affects the results cannot be ruled out. Besides, the dynamic changes of fat mass or lean tissues in the process of weight gain cannot be distinguished by the BMI values although BMI is widely used to assess excess adiposity and shows high test-retest reliability, it does not distinguish between increased fat mass and increased lean tissue mass, and hence can lead to significant misclassification (Prentice and Jebb, 2001). Moreover, the small sample size and motion artifacts during fMRI scanning may challenge the sensitivity and reliability of the current findings, longitudinal studies with larger sample size are warranted for looking into the structural and functional neural markers of weight change. Moreover, fMRI tasks such as the food odor Go/No-Go paradigm could further explore the neural predictors for future weight gain during specific processing of food odors (e.g., motivation or decision-making).

## Conclusion

In sum, results from the current study suggest that 2-year BMI increase was associated with lower baseline brain activation of the precuneus and PCC in response to high-fat vs. low-fat food odor, and lower activation of the SMA in response to food vs. non-food odor. This research shows that there are long-term consequences of body weight changes in selective responsivity to odorous food cues.

## Data Availability Statement

The raw data supporting the conclusions of this article will be made available by the authors, without undue reservation.

## Ethics Statement

The studies involving human participants were reviewed and approved by Ethics Board of the University of Dresden Medical School (#EK22012018). The patients/participants provided their written informed consent to participate in this study.

## Author Contributions

PH designed the study, performed the experiment, analyzed the data, and wrote the manuscript. TH and HC reviewed and revised the manuscript critically. All authors contributed to the article and approved the submitted version.

## Conflict of Interest

The authors declare that the research was conducted in the absence of any commercial or financial relationships that could be construed as a potential conflict of interest.

## Footnotes

^1^http://www.restfmri.net/forum/REST_V1.7
